# As Disparidades por Trás da Queda da Mortalidade Cardiovascular no Brasil

**DOI:** 10.36660/abc.20260615

**Published:** 2026-09-25

**Authors:** Aline F. Pedroso

**Affiliations:** 1 Section of Cardiovascular Medicine Department of Internal Medicine Yale School of Medicine New Haven CT EUA Section of Cardiovascular Medicine, Department of Internal Medicine, Yale School of Medicine, New Haven, CT – EUA; 2 Cardiovascular Data Science Lab Department of Internal Medicine Yale School of Medicine New Haven CT EUA Cardiovascular Data Science Lab, Department of Internal Medicine, Yale School of Medicine, New Haven, CT – EUA

**Keywords:** Doenças Cardiovasculares, Mortalidade, Desigualdades em Saúde, Brasil, Epidemiologia, Fatores Socioeconômicos

A mortalidade cardiovascular no Brasil diminuiu nas últimas décadas, uma tendência geralmente atribuída ao melhor controle dos fatores de risco cardiovascular e à ampliação do acesso aos serviços de saúde.^1,2^ No entanto, em um país marcado por profunda heterogeneidade geográfica e social, as médias nacionais podem mascarar diferenças substanciais no ritmo do progresso cardiovascular entre as regiões.^1,3^ Nesta edição dos Arquivos Brasileiros de Cardiologia, Saraiva et al. analisaram quase cinco milhões de mortes cardiovasculares registradas entre 2010 e 2023.^4^ Utilizando dados de mortalidade padronizados por idade do Sistema Nacional de Informações sobre Mortalidade e regressão de pontos de inflexão, os autores avaliaram as tendências nacionais, regionais e estaduais por sexo, incluindo mortalidade prematura e óbitos por doença isquêmica do coração, doença cerebrovascular e doença hipertensiva. A principal contribuição do estudo não é simplesmente confirmar a redução da mortalidade cardiovascular, mas também demonstrar a natureza desigual da transição cardiovascular no Brasil.^4^

O estudo apresenta diversas observações importantes. Primeiramente, a mortalidade cardiovascular geral diminuiu em nível nacional, tanto em homens quanto em mulheres, com as maiores reduções observadas, em geral, no Sul e Sudeste. Contudo, essas tendências favoráveis coexistiram com o aumento da mortalidade no Amapá entre ambos os sexos e no Amazonas entre os homens. Os resultados não devem ser reduzidos a um simples contraste entre um Sul e Sudeste mais ricos e um Norte e Nordeste menos favorecidos. Estados como Rondônia e Tocantins apresentaram tendências de melhora em alguns subgrupos demográficos, enquanto diferenças importantes também foram observadas dentro das regiões mais ricas. Em vez disso, o estudo demonstra que as classificações regionais são muito amplas para captar a organização local da prevenção e do atendimento cardiovascular.^4^

Essas diferenças são consistentes com o histórico mais longo de desigualdades no Brasil. Estudos anteriores associaram menor mortalidade cardiovascular a menor vulnerabilidade social, incluindo maior renda e escolaridade, melhor infraestrutura urbana e maior disponibilidade de serviços de saúde.^5-8^ O elevado número de mortes entre pessoas com pouca escolaridade formal é, portanto, importante, uma vez que a baixa escolaridade pode refletir desvantagens acumuladas ao longo da vida, incluindo diferenças nas condições de vida, conhecimento sobre saúde, exposições ocupacionais, acesso a cuidados preventivos e continuidade do tratamento.^9^ No entanto, essa descoberta deve ser interpretada com cautela. O estudo descreve a distribuição educacional das mortes cardiovasculares, em vez de taxas de mortalidade padronizadas por idade e nível de escolaridade. Portanto, identifica uma dimensão importante para futuras investigações, mas não quantifica o efeito independente da escolaridade sobre a mortalidade.

Outra descoberta importante do estudo é a variação nas tendências entre as causas cardiovasculares. A mortalidade cerebrovascular diminuiu amplamente em todas as regiões, estados e em ambos os sexos. As tendências na doença isquêmica do coração, no entanto, foram mais heterogêneas, incluindo aumento da mortalidade entre homens em nível nacional e padrões particularmente desfavoráveis em partes do Norte. A mortalidade por doença hipertensiva permaneceu estável em nível nacional, mas aumentou em vários estados, incluindo Amazonas, Bahia e Rio Grande do Sul. A mortalidade prematura por doença hipertensiva também aumentou entre homens em estados ricos, incluindo aqueles no Sul.^4^ Uma possível interpretação é que o sistema de saúde se tornou mais eficaz na prevenção de mortes após eventos cardiovasculares graves do que na prevenção da ocorrência desses eventos. Protocolos de emergência organizados, farmacoterapia baseada em evidências, reperfusão e prevenção secundária podem reduzir a mortalidade por acidente vascular cerebral e doença cardíaca isquêmica, mesmo quando o controle dos fatores de risco em nível populacional permanece incompleto.^10-12^ Em contrapartida, a mortalidade persistente ou crescente atribuída à doença hipertensiva pode indicar fragilidades em etapas anteriores do processo de cuidado, incluindo detecção, intensificação do tratamento e controle sustentado da pressão arterial. A hipertensão continua sendo o principal fator de risco cardiovascular modificável em todo o mundo, porém as taxas de controle permanecem inadequadas em muitos contextos.^13^

Essa interpretação, contudo, exige cautela. Óbitos codificados como doença hipertensiva são um indicador imperfeito do controle da pressão arterial, e diferenças nas práticas diagnósticas e na certificação da causa da morte podem influenciar os padrões observados. Ainda assim, a divergência entre a queda na mortalidade cerebrovascular e a estabilidade ou o aumento da mortalidade por doença hipertensiva é difícil de ignorar. Isso sugere que o acesso ao tratamento, por si só, é insuficiente. A ampla disponibilidade de medicamentos anti-hipertensivos no Brasil constitui uma base importante, mas a disponibilidade de medicamentos não garante a prescrição adequada, a adesão ao tratamento, ou o controle da pressão arterial.^14^ O modelo HEARTS aborda essas lacunas de implementação por meio de medição validada da pressão arterial, protocolos de tratamento padronizados, atendimento em equipe, fornecimento confiável de medicamentos e monitoramento contínuo do desempenho.^15^ As conclusões de Saraiva et al. reforçam a importância de avaliar se esses componentes são implementados de forma consistente em todos os territórios, em vez de considerar a adoção formal do programa como medida de sucesso.^4^

Os resultados específicos por sexo e sobre mortalidade prematura identificam populações para as quais o progresso tem sido particularmente limitado. Os homens representaram mais mortes cardiovasculares e, em geral, apresentaram reduções menores na mortalidade prematura do que as mulheres. As mulheres apresentaram declínios mais favoráveis na maioria das causas e regiões, embora tenha persistido uma heterogeneidade geográfica substancial.^4^ Essas diferenças podem refletir variações na exposição a fatores de risco, uso de cuidados preventivos, adesão ao tratamento e continuidade do cuidado. No entanto, o estudo não inclui os dados clínicos ou comportamentais em nível individual necessários para distinguir entre essas possíveis explicações.^9^

Alguns aspectos adicionais merecem consideração. O estudo é ecológico e descritivo. As análises de pontos de inflexão são importantes para identificar onde e quando as trajetórias de mortalidade mudaram, mas não podem determinar por que essas mudanças ocorreram. Portanto, seria inadequado atribuir tendências favoráveis ou desfavoráveis diretamente a uma política, programa ou intervenção específica. O período do estudo também inclui a pandemia de COVID-19, que interrompeu o acesso aos cuidados de rotina, alterou os padrões de hospitalização e pode ter afetado a certificação das causas básicas de óbito. A variação geográfica na qualidade dos dados de mortalidade apresenta outro desafio interpretativo. Regiões historicamente carentes de serviços de saúde têm apresentado melhorias no registro de óbitos, reduções em causas mal definidas e melhor identificação das causas básicas de óbito.^4^ Essas melhorias podem aumentar a mortalidade cardiovascular registrada mesmo sem um aumento correspondente no risco subjacente de morte. No entanto, é improvável que o aprimoramento do registro explique todo o padrão, principalmente quando tendências desfavoráveis persistem entre os sexos, em análises padronizadas por idade e em múltiplas categorias cardiovasculares.

O Brasil não vivencia uma única transição cardiovascular. São múltiplas transições em curso, em velocidades diferentes e, em alguns lugares, em direções distintas. A redução nacional da mortalidade cardiovascular é uma conquista importante, mas não pode ser o único parâmetro para medir o sucesso. A próxima fase da política cardiovascular deve ser avaliada pela sua capacidade de alcançar os estados e as populações onde o progresso tem sido mais lento. A medida mais significativa da continuidade da melhoria não será a magnitude da queda da média nacional, mas sim a rapidez com que as disparidades geográficas e sociais diminuem.
