# Supplementary material for: As Disparidades por Trás da Queda da Mortalidade Cardiovascular no Brasil
Source: Arq Bras Cardiol. 2026 Sep 25;123(8):e20260615. [Article in Portuguese] doi: 10.36660/abc.20260615 (PMC13668375; doi:10.36660/abc.20260615)
Supplement: Translation

# Translation: The Disparities behind Brazil’s Declining Cardiovascular Mortality

**DOI:** 10.36660/abc.20260615i

**Authors:** Aline F. Pedroso

**Affiliations:** 1 USA Section of Cardiovascular Medicine, Department of Internal Medicine, Yale School of Medicine, New Haven, CT – USA; 2 USA Cardiovascular Data Science Lab, Department of Internal Medicine, Yale School of Medicine, New Haven, CT – USA

**Keywords:** Cardiovascular Diseases, Mortality, Health Disparities, Brazil, Epidemiology, Socioeconomic Factors

Cardiovascular mortality in Brazil has declined over recent decades, a trend generally attributed to improved control of cardiovascular risk factors and expanded access to healthcare.^1,2^ Yet, in a country marked by profound geographic and social heterogeneity, national averages can obscure substantial differences in the pace of cardiovascular progress across regions.^1,3^ In this issue of Arquivos Brasileiros de Cardiologia, Saraiva et al. analyzed almost five million cardiovascular deaths recorded between 2010 and 2023.^4^ Using age-standardized mortality data from the national Mortality Information System and joinpoint regression, the authors evaluated national, regional, and state-level trends by sex, including premature mortality and deaths from ischemic heart disease, cerebrovascular disease, and hypertensive disease. The central contribution of the study is not simply confirming that cardiovascular mortality has declined, but also demonstrating the uneven nature of Brazil’s cardiovascular transition.^4^

The study has several important observations. First, overall cardiovascular mortality declined nationally in both men and women, with the largest reductions generally observed in the South and Southeast. However, these favorable trends coexisted with increasing mortality in Amapá among both sexes and in Amazonas among men. The findings should not be reduced to a simple contrast between a wealthier South and Southeast and an underserved North and Northeast. States such as Rondônia and Tocantins experienced improving trends among some demographic subgroups, while important differences were also observed within wealthier regions. Rather, the study demonstrates that regional labels are too broad to capture the local organization of cardiovascular prevention and care.^4^

These differences are consistent with the longer history of inequalities in Brazil. Previous studies have linked lower cardiovascular mortality to lower social vulnerability, including higher income and educational attainment, better urban infrastructure, and greater availability of health services.^5-8^ The large number of deaths among people with little formal education is therefore important, as low education can reflect accumulated disadvantage across the life course, including differences in living conditions, health literacy, occupational exposures, access to preventive care, and continuity of treatment.^9^ However, this finding should be interpreted with caution. The study describes the educational distribution of cardiovascular deaths rather than education-specific, age-standardized mortality rates. It therefore identifies an important dimension for further investigation but does not quantify the independent effect of education on mortality.

Another important finding of the study is the variation in trends across cardiovascular causes. Cerebrovascular mortality declined broadly across regions, states, and both sexes. Trends in ischemic heart disease, however, were more heterogeneous, including increasing mortality among men nationally and particularly unfavorable patterns in parts of the North. Hypertensive-disease mortality remained stable nationally but increased in several states, including Amazonas, Bahia, and Rio Grande do Sul. Premature mortality from hypertensive disease also increased among men in wealthy states, including those in the South.^4^ One possible interpretation is that the health system has become more successful at preventing death after major cardiovascular events than at preventing those events from happening. Organized emergency pathways, evidence-based pharmacotherapy, reperfusion, and secondary prevention can reduce mortality from stroke and ischemic heart disease even when population-level risk-factor control remains incomplete.^10-12^ In contrast, persistent or increasing mortality attributed to hypertensive disease may indicate weaknesses earlier in the care continuum, including detection, treatment intensification, and sustained blood-pressure control. Hypertension remains the leading modifiable cardiovascular risk factor globally, yet control rates remain inadequate in many settings.^13^

This interpretation nevertheless requires caution. Deaths coded as hypertensive disease are an imperfect proxy for blood-pressure control, and differences in diagnostic practices and cause-of-death certification may influence the observed patterns. Still, the divergence between declining cerebrovascular mortality and stable or increasing hypertensive-disease mortality is difficult to ignore. It suggests that access to treatment alone is insufficient. Brazil’s broad availability of antihypertensive medications provides an important foundation, but medication availability does not ensure appropriate prescribing, adherence, treatment intensification, or control.^14^ The HEARTS model addresses these implementation gaps through validated blood-pressure measurement, standardized treatment protocols, team-based care, reliable medication supply, and continuous performance monitoring.^15^ The findings by Saraiva et al. reinforce the importance of evaluating whether these components are consistently delivered across territories, rather than considering formal program adoption as an endpoint itself.^4^

The sex-specific and premature-mortality findings identify populations for whom progress has been particularly limited. Men accounted for more cardiovascular deaths and generally experienced smaller reductions in premature mortality than women. Women had more favorable declines across most causes and regions, although substantial geographic heterogeneity remained.^4^ These differences may reflect variation in risk-factor exposure, preventive care use, treatment adherence, and continuity of care. However, the study does not include the individual-level clinical or behavioral data needed to distinguish among these possible explanations.^9^

Some additional aspects merit consideration. The study is ecological and descriptive. Joinpoint analyses are valuable for identifying where and when mortality trajectories changed, but they cannot determine why those changes occurred. It would therefore be inappropriate to attribute favorable or unfavorable trends directly to a particular policy, program, or intervention. The study period also includes the COVID-19 pandemic, which disrupted access to routine care, altered patterns of hospitalization, and may have affected the certification of underlying causes of death. The geographic variation in mortality-data quality presents another interpretive challenge. Historically underserved regions have experienced improvements in death registration, reductions in ill-defined causes, and better identification of underlying causes.^4^ These improvements can increase recorded cardiovascular mortality even without a corresponding increase in the underlying risk of death. However, improved registration is unlikely to explain the full pattern, particularly when unfavorable trends persist across sexes, age-standardized analyses, and multiple cardiovascular categories.

Brazil does not have a single cardiovascular transition. It has multiple transitions unfolding at different speeds and, in some places, in different directions. The national decline in cardiovascular mortality is an important achievement, but it cannot be the endpoint against which success is measured. The next phase of cardiovascular policy must be evaluated by whether it reaches the states and populations in which progress has been slowest. The most meaningful measure of continued improvement will not be how far the national average falls, but how rapidly the geographic and social gaps beneath that average narrow.
